# Inhibition of Androgen Receptor Expression with Small Interfering RNA Enhances Cancer Cell Apoptosis by Suppressing Survival Factors in Androgen Insensitive, Late Stage LNCaP Cells

**DOI:** 10.1155/2013/519397

**Published:** 2013-02-06

**Authors:** Sang Soo Kim, Hee Joo Cho, Jung Yoon Kang, Hee Kyu Kang, Tag Keun Yoo

**Affiliations:** ^1^Eulji Medi-Bio Research Institute, Seoul, Republic of Korea; ^2^Department of Urology, College of Medicine, Eulji-University, Hangeulbiseok-gil, Hagye-dong, Nowon-ku, Seoul, Republic of Korea; ^3^Department of Biomedical Laboratory Science, College of Health Sciences, Eulji University, Gyeonggi-Do, Seongnam 461-713, Republic of Korea

## Abstract

*Introduction*.The aim was to evaluate the changes of androgen receptor (AR) expression quantitatively and to identify influence of AR on cancer related survival markers in LNCap cell line. *Materials and Methods*. We compared expressions of AR, heat shock protein 27 (HSP27), clusterin (CLU), glucose-related protein 78 (GRP78), and cellular FLICE-like inhibitory protein (c-FLIP) and their genes between es-LNCaP (less than 33 times subcultured, L-33), ls-LNCaP (over 81 times subcultured, H-81), and si-LNCaP (AR siRNA transfected ls-LNCaP) by Western blotting and RT-PCR. *Results*. The expressions of AR, HSP27, CLU, GRP78, and c-FLIP were increased in ls-LNCaP compared with es-LNCaP (AR, 157%; HSP27, 132%; CLU, 146%; GRP78, 138%; c-FLIP, 152%). However, in si-LNCaP cell line, protein expressions were reversed to the level of es-LNCaP cell lines (25, 102, 109, 98, and 101%), and gene expressions on real-time PCR were also reversed to the expression level of es-LNCaP (ls-LNCaP: 179, 156, 133, 123, and 167%; si-LNCaP: 22, 93, 103, 112, and 107%). *Conclusions*. This finding suggests that androgen receptor can be related to the increased expression of cancer related survival markers such as HSP27, GRP78, CLU, and c-FLIP in late stage prostate cancer, and also inhibition of AR gene can be a therapeutic target in this stage of cancer.

## 1. Introduction

Nearly 29% of patients with newly diagnosed cancer in United States were diagnosed with prostate cancer which is the second most common cause of cancer death (11%, 22,720 patients) [1, 2].

At early stage, the prostate cancer is influenced markedly by androgen acting through the androgen receptor (AR) and, clinically, could be treated with surgical castration, radiation, or antiandrogen therapy. However, after initial response to these treatments, most androgendependent prostate cancer cells commonly progress to a highly aggressive, metastatic, androgenindependent state.

Androgen receptor (AR) is a 110 kDa phosphoprotein and one of the nuclear receptor superfamily of ligand activated transcription factors which elicits the biological response of androgens [3–5]. The AR is expressed in nearly all prostate cancer cells [6–8]. Growth and development of aggressive prostate cancer depend on androgen induced AR function [9–11]. 

Androgen independent prostate cancer development can be explained by the following five theories. (1) the AR hypersensitivity: under chemical castrated state induced by androgen ablation treatment, more AR is produced or the AR has enhanced sensitivity to androgen. (2) The promiscuous AR hypothesis: factors other than testosterone (i.e., estrogens, progestins, and antiandrogens) acts as a mutated AR agonists due to broaden specificity of AR. (3) The outlaw AR hypothesis: hormone independent prostate cancer growth or progress of AR independently through PTEN mutation and activation of AR independent pathways such as PI3K and MAPK. (4) The bypass AR hypothesis: in androgen deprivation state, other antiapoptotic signal pathways through Bcl-2 overexpression and oncogene activation induce progression to HRPC. (5) The lurker cell hypothesis: androgen independent prostate cancer cells basically exist among epithelial stem cells even at androgen dependent state. After androgen deprivation, androgen independent malignant stem cells are selected to be activated [12].

LNCaP cell line is androgen sensitive human prostate cancer cells derived from the lymph node metastasis [13, 14]. Igawa et al. (2002) suggested the hormone sensitive LNCaP models changed to hormone independent cancer cells through long-term subcultures [15].

We focused on the factors related to the development of androgen independent prostate cancer. In previous studies using proteomic analysis, we confirmed that high passage subcultured LNCaP cells that acquired androgen independent property and the silencing of AR with small interfering RNA (siRNA) transfection resulted in the reversion of proteomic profile to level of es-LNCap cell line [16]. The aim of the present study was to evaluate changes of androgen receptor (AR) expression quantitatively and to identify influences of AR on cancer related proteins in LNCap cell line by comparing es-LNCaP and ls-LNCaP.

## 2. Materials and Methods

### 2.1. Cell Culture and Experimental Groups

LNCaP cells obtained from American Type Culture Collection (Bethesda, MD) were maintained in RPMI 1640 medium and made two LNCaP clones described in previous study [16]. All clones of LNCaP human prostate cancer cells were originated from the same source cell. The es-LNCaP cell was derived from low (less than 33) passage subculture and the ls-LNCaP, androgenindependent LNCaP, derived from high (more than 81) passage subculture. The si-ls-LNCaP subline was established by stably transfecting the ls-LNCaP cells with siRNA sequence. As control to silencing with siRNA, the scrambled siRNA, scr-ls-LNCaP was used.

### 2.2. Doxazosin and siRNA Treatment

Doxazosin (Sigma Aldrich Korea, Seoul, Korea) was prepared as described in previous study [17]. Cells were refed with fresh media at 80% confluence and treated with doxazosin or serum-free media containing 0.25% DMSO as control. The mRNA target sequences to AR (GeneBank Accession Number: NM000044) were designed using a siRNA template design tool (Ambion, Austin, TX), and siRNA was prepared with a Silencer siRNA construction kit (Ambion). Three oligonucleotides AR-1 (5′-GAC CUA CCG AGG AGC UUU CdTT-3′), AR-2 (5′-UCG AGG CCC UGU AAC UUG-3′), and AR-3 (5′-CAG UAG UUC GGA CAA ACG AAG A-3′) were designed based on the publicly released AR DNA sequence. The siRNAs were transfected into LNCaP cells with Lipofectamine 2000 (Invitrogen) employing 50 nM in 250 *μ*L OptiMEM medium/60 mm culture dish. The transfected cells were allowed to grow for 24, 48, and 72 h at 37°C in a 5% CO_2_ incubator and harvested for RT-PCR and immunoblot analysis. We performed immunohistochemical staining to confirm the expressions of AR in various LNCaP cells.

### 2.3. Total RNA Extraction, Conventional RT-PCR, and Real-Time RT-PCR

 Total RNA was extracted using the TRIzol method (Invitrogen, Carlsbad, CA). Cells (5.0 × 10^5^) were mixed in a test tube with 1 mL TRIzol solution. Prepared RNA was denaturated at 65°C for 15 min in a volume of 30 *μ*L and cooled on ice for at least 1 min. 2.0 *μ*g of denatured RNA were then annealed by addition of reaction mixture to a total volume of 20 *μ*L (4.0 *μ*L of 5 × RT buffer, 10 pmol of primers, 2.0 *μ*L of 25 mM MgCl_2_, 2.0 *μ*L of 10 mM dNTPs, and 0.2 *μ*L of 1 M DTT in nuclease-free water) and incubated at 42°C for 70 min. The reaction was terminated at 95°C for 5 min, chilled on ice for 5 min, and collected by brief centrifugation. To remove RNA, 1 *μ*L of RNase H were added to each tube followed by incubation at 37°C for 20 min. 1 *μ*L of cDNA were used for each PCR reaction.

Amplifications of cDNAs by PCR using specific primer pairs for AR were performed in 20 *μ*L reaction volumes containing 10 mM Tris-HCl, pH 8.3, 50 mM KCl, 1.5 mM MgCl_2_, 0.001% gelatin, 0.2 *μ*M each dNTP, 0.2 *μ*M of each primer, 1 unit of Taq DNA polymerase (Invitrogen, CA), and 1.0 *μ*L cDNA as template.

Real-time PCR was performed with an SLAN real-time PCR detection system (LG Life science, Korea) and SYBR Green reagents (Invitrogen, Carlsbad, CA). Specific primers for human GAPDH, AR, HSP27, CLU, GRP78, and c-FLIP were designed to work in the same cycling conditions (50°C for 2 min to permit uracil N-glycosylase cleavage, 95°C for 10 min, followed by 40 cycles of 95°C for 15 s, and 60°C for 1 min). We used 1.0 *μ*L of the reverse transcriptase product for PCR in a final volume of 25 *μ*L. 

### 2.4. Western Blot

Preparation of total cell lysate and the procedures for Western blot analyses were performed essentially as described previously [16]. The antibodies against GRP78, c-FLIP, and AR were purchased from Santa Cruz Biotechnology (Santa Cruz, CA). Antibody for HSP27 purchased from Millipore (Millipore, MA). The quantity of the applied protein was normalized with anti-actin polyclonal antibody (Sigma Aldrich Korea, Seoul, Korea).

Samples with equal amounts of protein (20 *μ*g) from lysates of cultured LNCaP cells were subjected to SDS-PAGE and then transferred to a PVDF filter. The filters were blocked in TBS containing 5% nonfat milk powder at 4°C overnight and then incubated for 1 h with a diluted each primary antibodies (Actin: 1 : 10,000; AR, HSP27, CLU, GRP78 : 1 : 1,000; c-FLIP: 1 : 2,000; Santa Cruz, CA). 

### 2.5. Immunocytochemical Analysis and TUNEL Staining

Cells on coverslips were rinsed 1 × phosphate-buffered saline (PBS) and then fixed with ice-cold methanol for 15 min. Samples were further permeabilized with PBS containing 0.025% Triton-X detergent (1 × PBS-TX) for 10 min and blocked with 3% BSA in 1 × PBS for 30 min. Cells were reacted with primary antibodies (AR, HSP27, CLU, GRP78 : 1 : 100; c-FLIP: 1 : 50; Santa Cruz, CA) for 1 hour at room temperature. Cells were washed 3 times for 5 min with 1 × PBS-TX and then incubated with horseradish peroxidase (HRP) conjugated secondary antibodies (goat anti-mouse IgG and goat anti-rabbit IgG, Santa Cruz. CA). Diaminobenzidine (DAB) was used as the chromogen and counterstaining was done with Mayers hematoxylin. Following three 5 min washes, cellscoverslips were mounted on slides with coverslips.

For TUNEL assays, fixed cells were incubated with an equilibrium buffer for 5 min using the in situ apoptosis detection kit, Fluorescein (Apoptag; Roche, BMS), and then treated in reaction buffer with 10 units of terminal deoxynucleotidyl transferase and 1 unit of deoxyuridine triphosphate digoxigenin at 37°C for 1 hour. The reaction was terminated by adding stop/wash buffer and then washed twice with Tris buffer. Anti-digoxigenin-FITC was added and reacted at 37°C for 30 min. After washing with distilled water, nuclei were counterstained with Hoechst 33258 (Sigma Chemical, St Louis, MO), and apoptosis in the cells was observed under a fluorescent microscope. Cells with green fluorescent (FITC) colored nuclei were considered apoptotic. For quantifying apoptotic cells, apoptotic and total cells were counted in 5 random fields scoring between 300 and 500 cells, and the numbers of apoptotic cells were expressed as percentages of the total cell population. Immunocytochemical staining slides and TUNEL staining slides were observed with microscope (TE-300, Nikon, Japan).

### 2.6. Statistics Analysis

Results were analyzed using a two-tailed Student's *t*-test to assess statistical significance. Values of *P* < 0.05 were considered statistically significant.

## 3. Results

### 3.1. Androgen Receptor siRNA Transfection Efficiency

**Figure 1:**
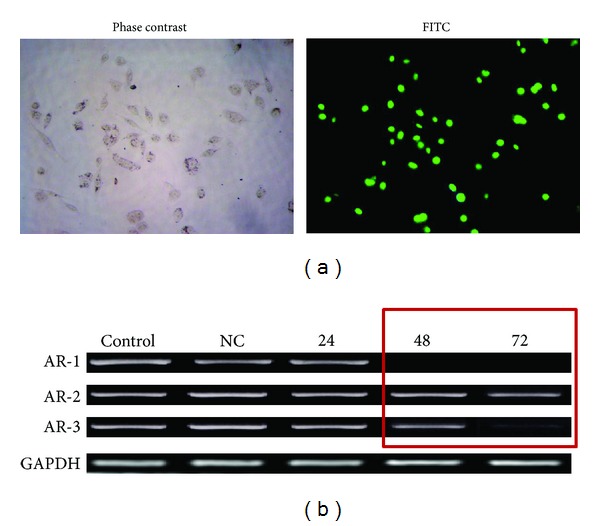
Effective silencing of the androgen receptor (AR) gene expression in ls-LNCap cells after small interfereing RNA (siRNA) treatment. (a) Transfection efficiency shown by fluorescence microscopy; (b) RT-PCR band of AR from si-ls-LNCap cells after siRNA. CNTL, untreated siRNA; NC, treated scrambled siRNA.

 To explore the feasibility of siRNA transfection efficiency in knocking down AR expression in prostate cancer cells that harbor the AR gene, fluorescent oligo staining with BLOCK-iT was used and the green staining of more than 95% of cells was confirmed in siRNA transfected cells under fluorescence microscopy (Figure 1(a)). We designed and synthesized three siRNAs, AR-1, AR-2, and AR-3, against human AR gene. After 48 hours transfection with three sequence-specific siRNAs, one relatively potent siRNA, AR-1, was identified in knocking down AR expression compared with others by checking the mRNA with RT-PCR. This knocking down effect was sequence-specific event because a negative control siRNA with a scrambled sequence had no effect on AR expression level (Figure 1(b)).

### 3.2. Immunocytochemistry of Markers

**Figure 2:**
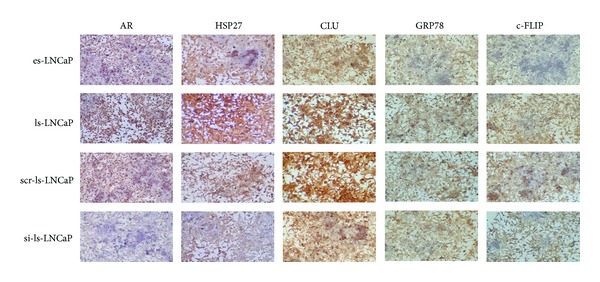
Immunocytochemical analysis of HSP27, clusterin, GRP78, and c-FLIP at the es-LNCap, ls-LNCap, scr-ls-LNCap, and si-ls-LNCaP cells.

Among the four experimental cell lines (es-LNCaP, ls-LNCaP, scr-ls-LNCaP, and si-ls-LNCaP), we confirmed the expression level of five prostate cancer related proteins (AR, HSP27, CLU, GRP78, and c-FLIP) with immunocytochemical staining (Figure 2). Positive staining of AR protein was more intensive in ls-LNCaP than es-LNCaP, but the expression level of AR protein in si-ls-LNCaP was almost disappeared (Figure 2). These results showed AR gene expression was almost inhibited by siRNA transfection. Moreover, the knocking down effect on the other cancer related proteins was also verified similarly. The positive staining to HSP27, CLU, GRP78, and c-FLIP proteins in ls-LNCaP cells was more intensive than in es-LNCaP cells and was dramatically decreased in si-ls-LNCaP cells.

### 3.3. Gene and Protein Expressions of Markers

**Figure 3:**
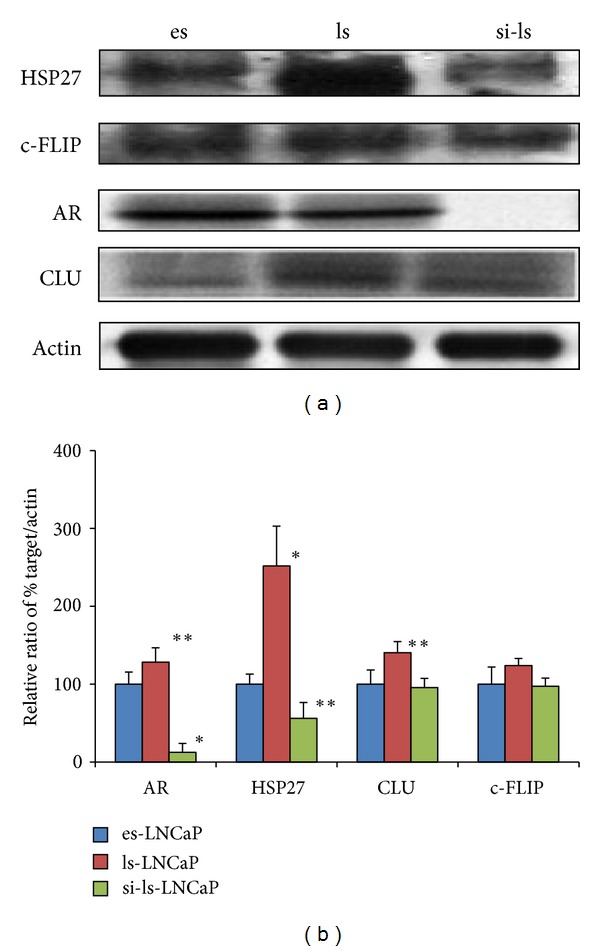
Electrophoretogram of immunoblot for androgen receptor, HSP27, c-FLIP, and clusterin expression at the es-, ls-, and the si-ls-LNCap cells.

**Figure 4:**
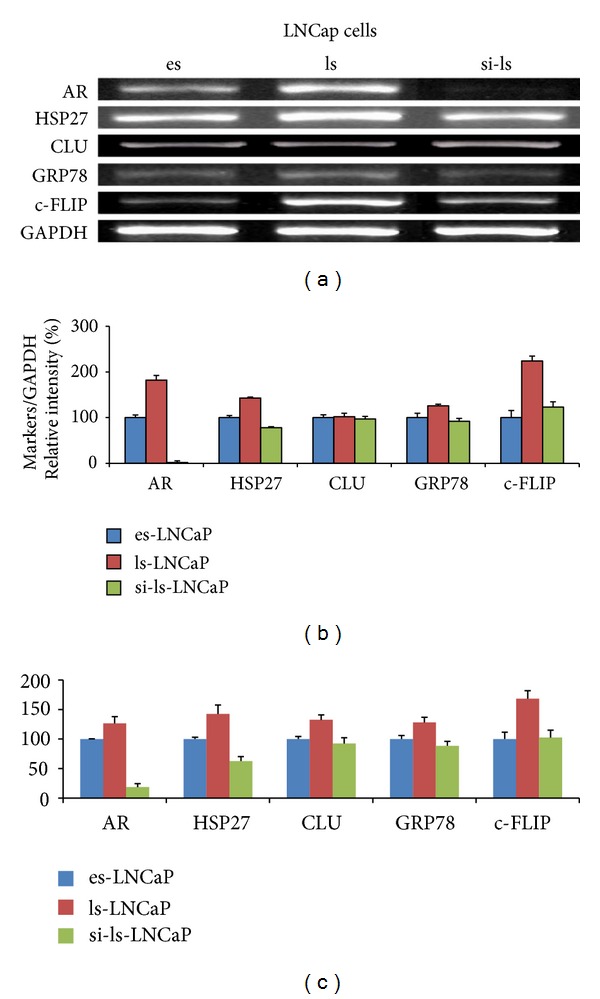
Electrophoretogram and its densitogram of conventional RT-PCR/real-time PCR product for androgen receptor, HSP27, CLU, GRP78, and c-FLIP expression at the es-, ls-, and the si-ls-LNCaP cells.

The expression of five prostate cancer related proteins (AR, HSP27, CLU, GRP78, and c-FLIP) increased in ls-LNCaP compared with es-LNCaP (AR, 157%; HSP27, 132%; CLU, 146%; GRP78, 138; and c-FLIP, 152%; Figure 3). But in si-ls-LNCaP cell line, protein expressions were decreased to level of es-LNCaP cell lines (25, 102, 109, 98, and 101%; Figure 3), and gene expressions on real-time PCR were decreased similarly (ls-LNCaP: 179, 156, 133, 123, and 167%; si-ls-LNCaP: 22, 93, 103, 112, and 107%; Figure 4).

### 3.4. TUNEL Assay

**Figure 5:**
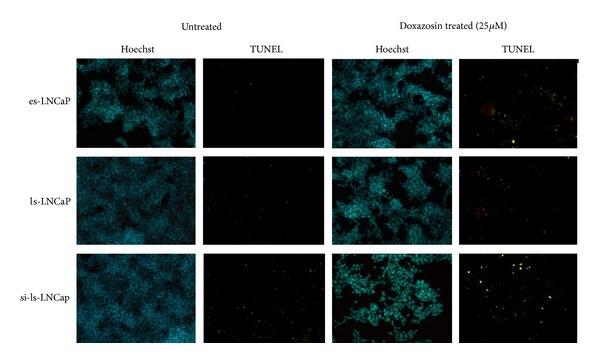
In situ detection of apoptotic cells in AR siRNA transfected cells at 48 hours after doxazosin treatment (25 *μ*M). In situ detection of apoptotic cells in LNCaP cells was performed by 3′-end labeling with digoxigenin-dUTP using terminal transferase.

TUNEL assay was performed to see how doxazosin induced apoptosis was affected by the inhibition of AR gene (Figure 5). The number of TUNEL positive cells appeared less in ls-LNCaP cells compared to es-LNCaP counterpart. But, after AR was silenced, the number of TUNEL positive cells increased significantly.

## 4. Discussion

Prostate cancer cells are basically androgen dependent and androgen deprivation therapy (ADT) consistently causes prostate apoptosis and involution in first diagnosed prostate cancer. But, when prostate cancer advance further, it progresses into a more aggressive form of castration resistant prostate cancer (CRPC), refractory to all kinds of ADT. Treatment of CRPC is very difficult and not that satisfactory so far. Docetaxel based chemotherapy is one of the most effective ways of treatments [18–20], but the overall survival benefit is only 2-3 months compared to conventional methods [21].

Diverse pathways have been discussed regarding progression to CRPC from androgen dependent counterpart. Among them, AR is considered having one of the most important roles with the possible mechanisms of hypersensitive AR or mutation of AR gene [12]. LNCaP cell lines are well known as their androgen sensitive characteristics, but in our previous study, we showed the changes of cellular characteristics of early stage LNCaP cells (L-33) into androgen independent manner after long-term subculture (H-81) [16]. And we also found that there were significant differences in the expression of important survival antiapoptotic factors such as Tim, GRP78, and HSP27 using proteomics technique. We think that early and late stage LNCaP cell line model can help us to understand the mechanism of progression into the form of CRPC to some extent. In the present study, we showed higher level of AR mRNA and protein expression in H-81 compared to L-33, and this implicates that AR is closely related to the progression into H-81 characteristics via direct or indirect ways. 

Among the possible mechanisms of chemotherapy failure of CRPC, several antiapoptotic factors such as c-FLIP [22], HSP27 [23], clusterin [17], and GRP78 [24] have been discussed. These factors have protective roles against apoptosis inducing stimuli. They are upregulated in various types of cancer cells and the degree of upregulation is proportional to the cancer aggressiveness. It also has been speculated that these survival factors help cancer cells to resist against various forms of anticancer treatment such as radiotherapy and cytotoxic chemotherapy. 

Hsp27 suppresses apoptosis and probably has a critical role in progression to CRPC [25–29]. It has been reported that androgen insensitive LNCaP cells showed upregulation of HSP27 against androgen withdrawal and antiacancer drugs, such as paclitaxel [30]. 

GRP78 is a key member of the molecular chaperone heat shock protein (HSP) 70 family [31–33]. GRP78 expression is increased when AR expression is upregulated in LNCaP cells treated with DHT [34]. This is consistent with our findings in this study, showing further upregulation of GRP78 expression in H-81 cells.

Clusterin acts as an antiapoptotic factor and plays an important role in resistance to chemotherapeutic drugs [35]. When clusterin is overexpressed using vector transfection in rat prostate cell lines, transfected cells survived with blocking TNF-*α* induced apoptosis.

c-FLIP is also involved in apoptosis pathway regarding Fas signal transduction [22]. It is generally considered to have antiapoptotic roles in the prostate cancer [36]. c-FLIP expression is highly upregulated in the prostate cancer tissue when compared to normal tissue. It seems that maintaining high level of c-FLIP is essential and important in overcoming TNF related apoptosis in the prostate cancer [37]. It has also been known that transcription of c-FLIP is affected by AR [38]. 

Our study showed increased expression of clusterin, HSP27, GRP78, and c-FLIP in H-81 compared to L-33. mRNAs and proteins of these factors are downregulated below the levels of L-33 after AR knock-out using siRNA technique. Furthermore, the same concentration of doxazosin could induce more significant apoptosis after AR silencing. These observations suggest that Clusterin, HSP27, GRP78, and c-FLIP take part in the progression into H-81 in LNCaP model and AR is closely related to the upregulated expression and suppression of these survival factors. Our study also helps us to speculate that while prostate cancer cells become more aggressive with change of AR and overproduction of survival factors under the extremely stressful circumstances like androgen deprivation, these cells can be reverted into treatment sensitive traits when successful AR blocking causes suppression of various survival factors. It also supports the idea that therapeutic approach targeting AR can enhance the efficacy of anticancer treatment in the patients with metastatic CRPC, resisting against all forms of treatment.

Suppression of gene expression using siRNA is a simplified experimental technique which can regulate functions of specific factors at the gene level. AR silencing in the gene level is essential in the study of AR block, because AR is a transcription factor related to synthesis, regulation, and secretion of various kinds of proteins. In our present study, AR silencing successfully showed over 80% efficiency. We also showed higher level of discrimination using real time RT-PCR which enables us to find more significant differences between cell groups.

It can be summarized that in this LNCaP model, we observed that c-FLIP, HSP27, clusterin, and GRP78 take part in the progression into androgen insensitive status and the existence and overexpression of AR are closely related in this process. We think that these findings can be applied in the understanding of CRPC progression and treatment resistance of metastatic CRPC. New therapeutic approaches targeting AR regulation could be an effective solution against metastatic CRPC, currently incurable, and therefore various scientific efforts should be focused.
